# Association between neutrophil percentage-to-albumin ratio and breast cancer in adult women in the US: findings from the NHANES

**DOI:** 10.3389/fnut.2025.1533636

**Published:** 2025-04-28

**Authors:** Huikai Liang, Kelun Pan, Jiayi Wang, Jianqing Lin

**Affiliations:** ^1^Department of Breast and Thyroid Surgery, The Second Affiliated Hospital of Fujian Medical University, Quanzhou, China; ^2^The Second Clinical College of Fujian Medical University, Quanzhou, China

**Keywords:** NHANES, inflammation, nutrition, breast cancer, NPAR

## Abstract

**Background:**

An increasing number of studies suggests an association between systemic inflammation, nutritional status, and cancer. However, the relationship between the prevalence of breast cancer (BC) and the neutrophil-percentage-to-albumin ratio (NPAR), a recently identified biomarker of inflammation, is not well established. Therefore, this study aims to investigate the relationship between BC risk and the NPAR.

**Methods:**

This study included 18,726 participants from the National Health and Nutrition Examination Survey (NHANES) conducted between 2001 and 2018. The NPAR was used to assess inflammation and nutritional status. Statistical methods such as multivariate logistic regression, subgroup analysis, and restricted cubic spline (RCS) analysis were conducted to investigate the influence of NPAR on the prevalence of BC. In addition, propensity score matching was employed to further validate the findings.

**Results:**

The logistic regression results showed that the prevalence of breast cancer is significantly associated with the NPAR (OR = 1.05; 95% CI = 1.02–1.09, *p* = 0.003). In comparison to participants in the lowest quartile, Q1, the prevalence of breast cancer increased by 5% for those in Q2 (*p* = 0.745), 3% for those in Q3 (*p* = 0.032), and 38% for those in Q4 (*p* = 0.018) with a higher NPAR. In addition, subgroup and RCS analyses showed that the NPAR and BC prevalence were positively correlated. Furthermore, a significant association was observed between the NPAR and marital status. The significance of traits was assessed using mean decrease accuracy (MDA) and mean decrease impurity (MDI). These measures of random forest modeling showed that NPAR is one of the major factors affecting the prevalence of BC. Furthermore, linear analysis demonstrated a correlation between a high NPAR and increased total testosterone and sex hormone-binding globulin (SHBG) levels.

**Conclusion:**

A significant association was observed between a high NPAR and a higher prevalence of breast cancer, which could be attributable to sex hormone levels. This finding suggests that the NPAR may serve as a biomarker for BC in adult women in the US.

## Introduction

Breast cancer (BC) has the highest mortality and incidence rates among women, posing a significant risk to women’s health worldwide. Based on global cancer statistics, BC accounts for 11.6% of all newly diagnosed cancer cases (1). It is a type of malignant tumor when the epithelial tissue of the breast undergoes uncontrolled growth and malignant changes. Several factors contribute to the development of this condition, including lifestyle, sex hormone levels, and inheritance, but its exact causes remain unknown (2).

Numerous studies have reported that inflammatory and nutritional markers can act as valuable indicators of cancer (3). As early as the 18th century, chronic inflammation was considered to be associated with the onset of cancer (4). Previous studies have shown that systemic inflammation may contribute to a higher incidence of BC (5). In addition, poor clinical outcomes in BC survivors are strongly associated with their nutritional habits and levels of inflammation (6). Currently, peripheral blood markers such as the lymphocyte-to-monocyte ratio and neutrophil-to-lymphocyte ratio (NLR) are commonly used to predict the prognosis of cancer (7, 8). Furthermore, studies have proposed that an appropriate diet can reduce the risk of cancer, especially colon cancer and BC, and that inadequate nutrition promotes the growth of cancer cells and increases the probability of acquiring the disease (9). The survival chances of BC patients can be predicted using two nutritional indices: the Controlled Nutritional Status (CONUT) and the Prognostic Nutritional Index (PNI) (10).

The neutrophil percentage-to-albumin ratio (NPAR) outperforms traditional inflammatory biomarkers by combining neutrophil (inflammatory) and albumin (nutritional) indicators. This combination provides greater stability and clinical utility. As an emerging biomarker, the NPAR not only measures inflammation and nutritional status but has also been linked to the prognosis of conditions such as depression, steatotic liver disease, and cardiovascular disease (CVD) (11–13). However, no study has investigated the association between BC prevalence and the NPAR. Using the data from the National Health and Nutrition Examination Survey (NHANES) conducted between 2001 and 2018, this study aims to explore the relationship between the NPAR and BC in US women.

## Materials and methods

### Study population

This study utilizes data from the NHANES database published between 2001 and 2018. The NHANES is a large-scale, continuous, biannual cross-sectional survey that includes demographic, nutritional, screening, laboratory, and questionnaire data, among other relevant details. The survey proposal was approved by the Research Ethics Review Board of the Centers for Disease Control and Prevention and the National Center for Health Statistics (NCHS).

**Figure 1:**
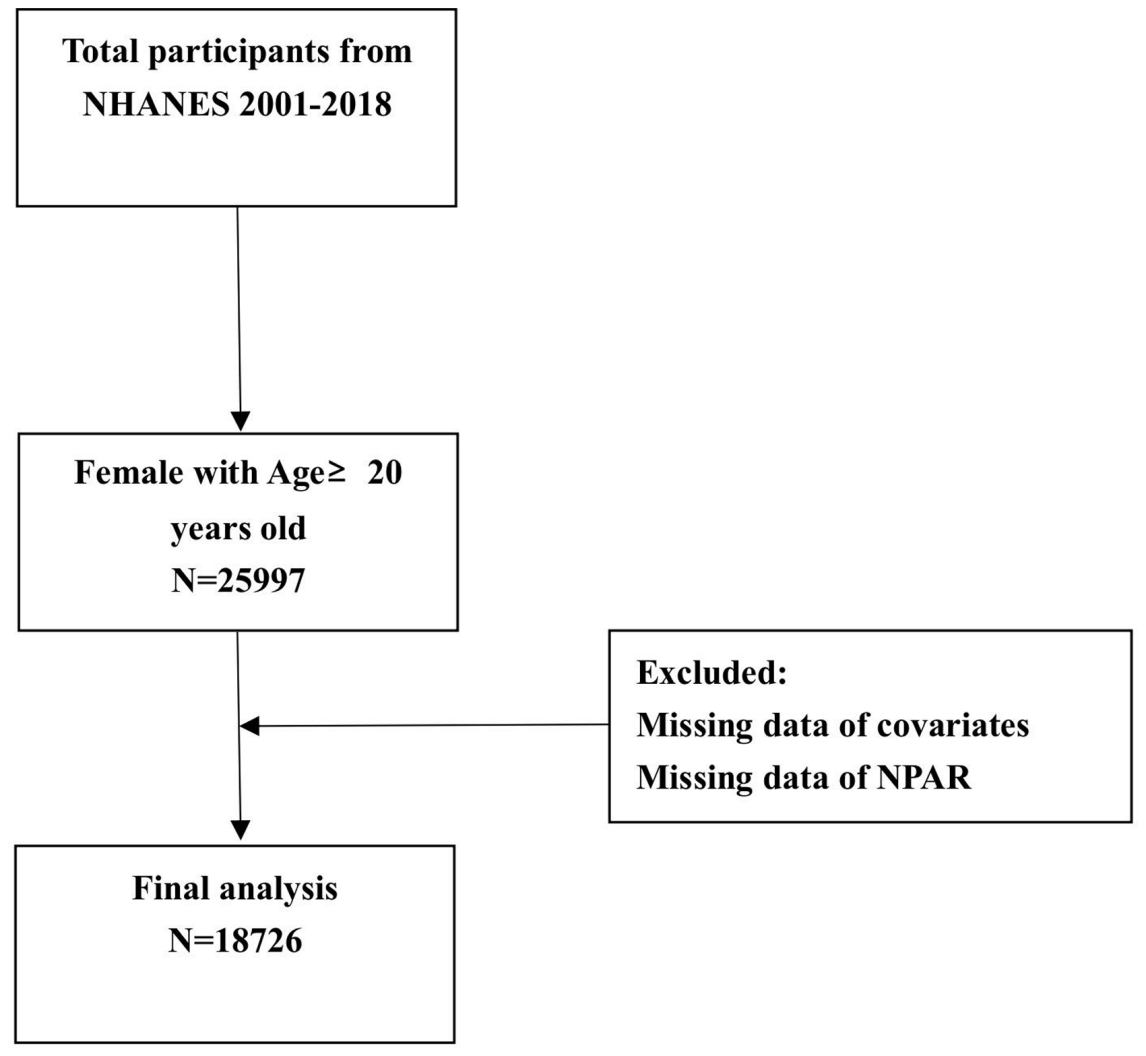
Flowchart of sample screening.

This study included data obtained from 25,997 female participants aged 20 years and above across nine cycles conducted between 2001 and 2018. In total, 18,726 participants were included in this study as 7,271 participants were excluded for missing data. The procedure used in the sample selection process is shown in Figure 1.

### Measurement of the NPAR

The NPAR, which refers to the ratio of neutrophil percentage to serum albumin levels, can be used to evaluate systemic inflammation and nutritional status. The formula for calculating NPAR is as follows: NPAR = neutrophil percentage (of total blood count) (%) × 100/albumin (g/dL).

### Assessment of breast cancer

A self-reported medical condition questionnaire was administered to obtain information about the diagnosis of BC. Patients were considered to have BC if a doctor had informed them of their diagnosis.

### Covariates

The following covariates were adjusted based on previous research experience: age, marital status (married/living with partner, widowed/divorced/separated, or never married), ethnicity (Mexican American, non-Hispanic Black, non-Hispanic White, other Hispanic, or other ethnicity), education level (less than high school, high school or equivalent, or college or above), BMI (<25 kg/m^2^, 25–30 kg/m^2^, or ≥30 kg/m^2^), cardiovascular disease (CVD) (yes or no), poverty-to-income ratio (PIR) (≤1.30, 1.30–3.50, or >3.50), alcohol drinking (no/<12 cups per year or at least 12 cups per year), smoking status (no/<100 cigarettes in life or at least 100 cigarettes in life), hypertension (yes or no), and diabetes (yes or no). CVD included heart attack, myocardial infarction, angina pectoris, coronary heart disease, and congestive heart failure based on the diagnosis by a physician. Participants who met one or more of the following conditions were diagnosed as having diabetes: (1) glycated hemoglobin (HbA1c) level ≥6.5%; (2) random blood glucose level ≥11.1 mmol/L; (3) fasting blood glucose level ≥7.0 mmol/L or 2-h oral glucose tolerance test level ≥11.1 mmol/L; and (4) self-reported medical diagnosis of diabetes. A history of hypertension, self-reported use of antihypertensive medication, a mean diastolic blood pressure of 90 mmHg, and a mean systolic blood pressure of 140 mmHg were considered the indicators of hypertension. As mentioned earlier, a self-reported medical condition questionnaire was used to identify BC diagnosis, and patient were considered to have BC if a doctor had diagnosed them with BC.

### Measurement of sex hormones

All data on sex hormone levels were retrieved from the NHANES database. The levels of estradiol (E2), serum total testosterone (TT), and sex hormone-binding globulin (SHBG) were directly measured using standardized NHANES laboratory protocols. Detailed experimental methodologies are available in the NHANES Laboratory Procedures Manual.

### Statistical analysis

Participants were divided into two groups for a comparison of baseline characteristics based on whether they had BC. Continuous variables were represented as medians (interquartile spacing, or IQR), whereas categorical variables were expressed as numbers and percentages. The baseline characteristics of continuous and categorical variables were compared using non-parametric and chi-squared tests, respectively. To evaluate the independent association between the NPAR and BC prevalence, multivariate logistic regression equations were used: model 1 did not adjust for covariates; model 2 adjusted for covariates, taking into account age, marital status, ethnicity, and education levels; and model 3 further adjusted for diabetes, hypertension, smoking status, alcohol drinking, BMI, CVD, and PIR. Restricted cubic spline (RCS) analysis was performed to investigate the non-linear relationship between the NPAR and BC. Then, applying a machine learning approach modeled using the random forest method, the relative importance of the influence of each factor on BC was examined. In addition, a sensitivity analysis study was carried out to ascertain the dependability of the results. To further validate the NPAR–BC association, all covariates were subjected to propensity score matching (PSM) at a ratio of 1:1 to ensure that the distribution of covariates was similar between the BC and non-BC groups. PSM was also used to analyze the relationship between NPAR and BC. To identify the heterogeneity of the association between BC and the NPAR and validate the generalizability of results, subgroup analyses were conducted. Furthermore, the associations between the NPAR and the levels of sex hormones (E2, TT, and SHBG) were investigated to further identify the potential pathways. All statistical analyses were conducted using R software (version 4.2.0). A *p* < 0.05 was considered statistically significant.

## Results

### Baseline characteristics

A total of 18,726 participants were included, with a mean age of 49.04 ± 18.04 years and a mean NPAR of 14.38 ± 3.03, of which 511 participants had BC. There were no statistically significant differences in education level, BMI, alcohol consumption between the two groups (*p* > 0.05); however, statistically significant differences (*p* < 0.05) were observed in age, NPAR, PIR, marital status, ethnicity, hypertension, diabetes, smoking, and CVD (Table 1).

**Table 1:** Baseline characteristics of participants by breast cancer status.

Variables	Total (*n* = 18,726)	Control (*n* = 18,215)	Breast cancer (*n* = 511)	*p*-value
Age, Mean ± SD	49.04 ± 18.04	48.53 ± 17.92	67.21 ± 11.83	**<.001**
NPAR, Mean ± SD	14.38 ± 3.03	14.37 ± 3.04	14.71 ± 2.81	**0.013**
Edu, *n* (%)				**0.480**
Less than high school	4,353 (23.25)	4,245 (23.30)	108 (21.14)	
High school or equivalent	4,191 (22.38)	4,077 (22.38)	114 (22.31)	
College or above	10,182 (54.37)	9,893 (54.31)	289 (56.56)	
PIR, *n* (%)				**<.001**
≤1.30	5,955 (31.80)	5,837 (32.05)	118 (23.09)	
1.30–3.50	7,124 (38.04)	6,914 (37.96)	210 (41.10)	
>3.50	5,647 (30.16)	5,464 (30.00)	183 (35.81)	
Marital status, *n* (%)				**<.001**
Married/Living with partner	10,413 (55.61)	10,156 (55.76)	257 (50.29)	
Widowed/Divorced/Separated	5,266 (28.12)	5,039 (27.66)	227 (44.42)	
Never married	3,047 (16.27)	3,020 (16.58)	27 (5.28)	
Race, *n* (%)				**<.001**
Mexican American	3,093 (16.52)	3,052 (16.76)	41 (8.02)	
Other Hispanic	1,568 (8.37)	1,539 (8.45)	29 (5.68)	
Non-Hispanic White	8,742 (46.68)	8,406 (46.15)	336 (65.75)	
Non-Hispanic Black	3,749 (20.02)	3,674 (20.17)	75 (14.68)	
Other Race	1,574 (8.41)	1,544 (8.48)	30 (5.87)	
Hypertension, *n* (%)				**<.001**
Yes	7,743 (41.35)	7,411 (40.69)	332 (64.97)	
No	10,983 (58.65)	10,804 (59.31)	179 (35.03)	
DM, *n* (%)				**<.001**
Yes	2,923 (15.61)	2,785 (15.29)	138 (27.01)	
No	15,803 (84.39)	15,430 (84.71)	373 (72.99)	
Smoking status, *n* (%)				**0.025**
At least 100 cigarettes in life	11,663 (62.28)	11,369 (62.42)	294 (57.53)	
NO/<100 cigarettes in life	7,063 (37.72)	6,846 (37.58)	217 (42.47)	
BMI, *n* (%)				**0.642**
< 25 kg/m^2^	5,709 (30.49)	5,548 (30.46)	161 (31.51)	
25–30 kg/m^2^	5,420 (28.94)	5,267 (28.92)	153 (29.94)	
≥ 30 kg/m^2^	7,597 (40.57)	7,400 (40.63)	197 (38.55)	
CVD, *n* (%)				**<.001**
Yes	1,282 (6.85)	1,208 (6.63)	74 (14.48)	
No	17,444 (93.15)	17,007 (93.37)	437 (85.52)	
Alcohol drinking, *n* (%)				**0.592**
No/<12 cups per year	3,890 (20.77)	3,779 (20.75)	111 (21.72)	
At least 12 cups per year	14,836 (79.23)	14,436 (79.25)	400 (78.28)	
NPAR quantile, *n* (%)				**<.001**
Q1	4,680 (24.99)	4,577 (25.13)	103 (20.16)	
Q2	4,673 (24.95)	4,566 (25.07)	107 (20.94)	
Q3	4,682 (25.00)	4,532 (24.88)	150 (29.35)	
Q4	4,691 (25.05)	4,540 (24.92)	151 (29.55)	

Bold values indicate statistically significant *p*-values (*p* < 0.05).

### Association between the NPAR and BC

The results of the logistic regression analysis of the association between the NPAR and BC prevalence are presented in Table 1. After controlling for relevant confounders, the NPAR was significantly correlated with BC (OR = 1.05, 95% CI = 1.02–1.09, *p* = 0.003). In addition, using the lowest quartile, Q1, as a reference, sensitivity analyses were carried out utilizing quartiles, and the risk of BC was higher in Q2–Q4 in model 3 (Q2: OR = 1.05, 95% CI: 0.79–1.39; Q3: OR = 1.33, 95% CI: 1.03–1.73; Q4: OR = 1.38, 95% CI: 1.06–1.79), and the prevalence of breast cancer was higher in Q2, Q3, and Q4 participants by 5% (*p* = 0.745), 3% (*p* = 0.032), and 38% (*p* = 0.018) compared to Q1 participants (Table 2).

**Table 2:** Association of NPAR and breast cancer among US participants, NHANES, 2001 to 2018.

NPAR	Model 1		Model 2		Model 3	
	OR	*p*-value	OR	*p*-value	OR	*p*-value
Continuous quantile	1.04 (1.01–1.06)	**0.013**	1.05 (1.02–1.09)	**0.001**	1.05 (1.02–1.09)	**0.003**
Q1	–	–	–	–	–	–
Q2	1.04 (0.79–1.37)	0.772	1.06 (0.80–1.40)	0.703	1.05 (0.79–1.39)	0.745
Q3	1.47 (1.14–1.90)	**0.003**	1.35 (1.04–1.76)	**0.024**	1.33 (1.03–1.73)	**0.032**
Q4	1.48 (1.15–1.90)	**0.003**	1.41 (1.08–1.83)	**0.011**	1.38 (1.06–1.79)	**0.018**

OR: Odds Ratio, CI: Confidence Interval. Model 1: Crude. Model 2: Adjust: age, education, marital status, race. Model 3: Adjust: age, education, marital status, race, PIR, hypertension, DM, smoking status, BMI CVD, and alcohol drinking. Bold values indicate statistically significant *p*-values (*p* < 0.05).

### Ranking of random forest model feature importance

**Figure 2:**
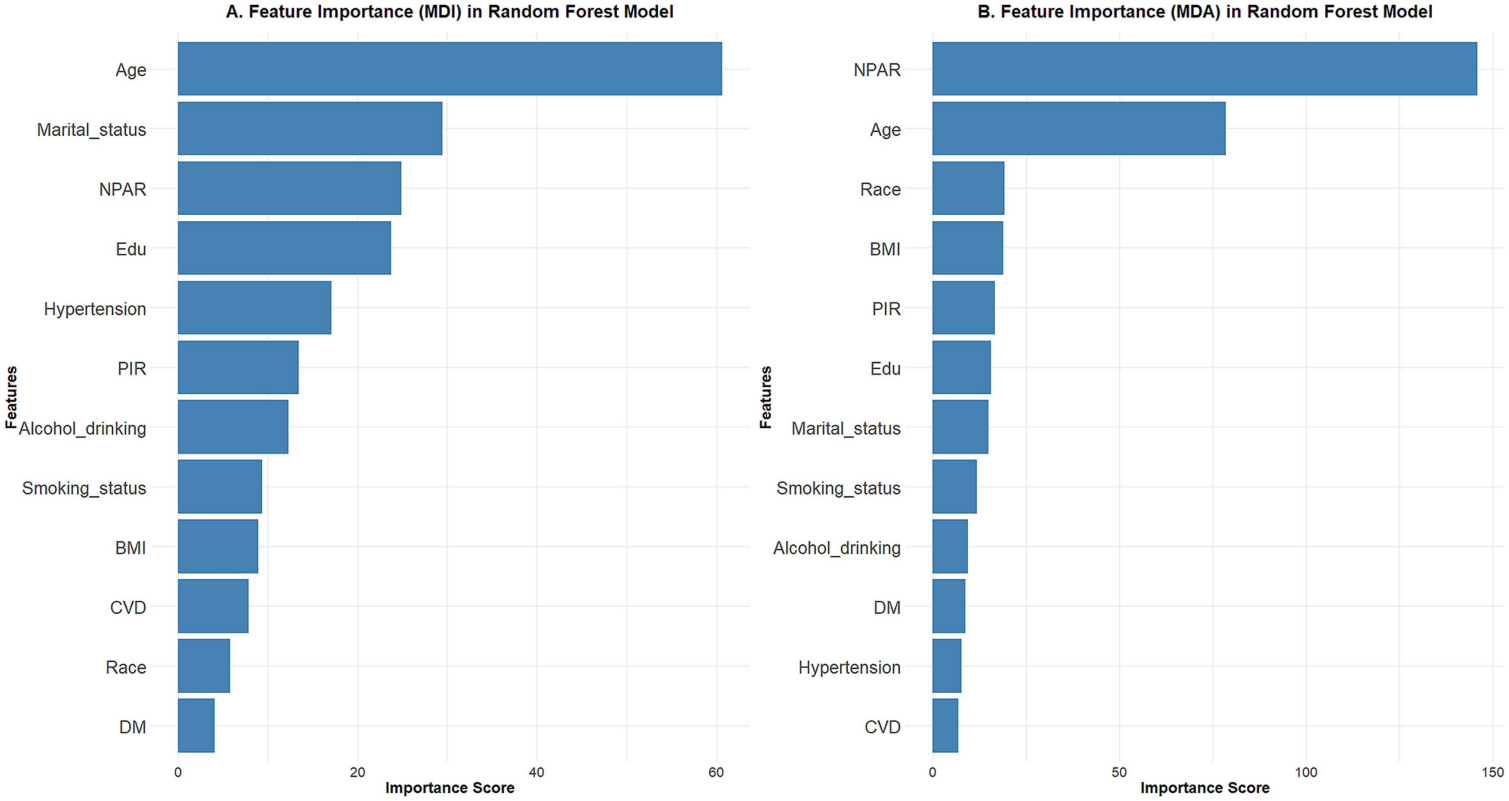
Using the random forest model to rank feature importance using different metrics. **(A)** Mean decrease impurity; **(B)** mean decrease accuracy. The results showed that the NPAR is one of the important factors in the development of breast cancer.

We used two measures of feature importance ranking of the random forest model to forecast the relative relevance of the included variables, namely mean decrease accuracy and mean decrease impurity. The results showed that the NPAR was a significant determinant of BC prevalence in both metrics (Figure 2).

### Exploring nonlinear relationships

**Figure 3:**
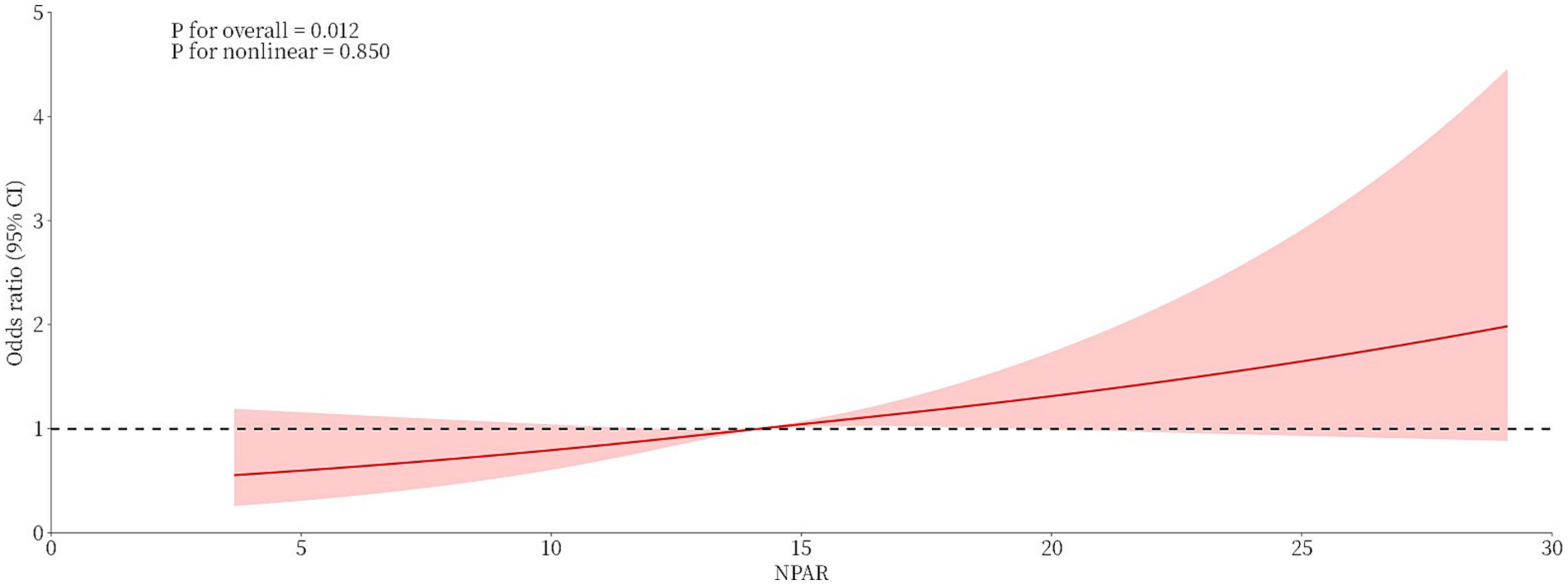
Linear association between the NPAR and breast cancer prevalence based on restricted cubic spline analysis.

RCS curves were used to investigate the nonlinear relationship between the NPAR and BC prevalence, and the fully adjusted model suggested no nonlinear association between the NPAR and BC prevalence (*P* for nonlinearity = 0.850). The findings that the OR increased as NPAR values increased and that, for the majority of ranges, the confidence intervals did not contain 1 showed a statistically significant positive linear association between the NPAR and BC prevalence (Figure 3).

### Sensitivity analysis

First, PSM was conducted, and of the 1,020 participants, 510 had BC and 510 were not diagnosed with BC (Supplementary Table S1). After controlling for differences in the variables between the two groups (*p* > 0.05), another logistic regression analysis was carried out, and this time, the NPAR correlated significantly with BC (OR = 1.10, 95% CI 1.05–1.16, *p* < 0.001). Second, subgroup analyses were carried out for the variables such as age, education level, marital status, PIR, ethnicity, diabetes, hypertension, BMI, smoking status, alcohol drinking, and CVD. The findings showed that there was a consistent positive correlation between the NPAR and BC among all subgroups. Additionally, the positive correlation remained stable for most of the population, though marital status significantly influenced this relationship (interaction effect *P* = 0.012) (Table 3).

**Table 3:** Subgroup analysis of the association between NPAR and breast cancer.

Variables	*n* (%)	OR (95%CI)	*p*-value	*P* for interaction
All patients	18,726 (100.00)	1.08 (1.04–1.11)	**<0.001**	
Edu				0.452
Less than high school	4,353 (23.25)	1.11 (1.03–1.18)	**0.003**	
High school or equivalent	4,191 (22.38)	1.09 (1.02–1.17)	**0.015**	
College or above	10,182 (54.37)	1.06 (1.01–1.11)	**0.009**	
PIR				0.834
≤1.30	5,955 (31.80)	1.09 (1.02–1.16)	**0.011**	
1.30–3.50	7,124 (38.04)	1.07 (1.01–1.13)	**0.012**	
> 3.50	5,647 (30.16)	1.08 (1.02–1.14)	**0.006**	
Marital status				0.012
Married/Living with partner	10,413 (55.61)	1.03 (0.98–1.08)	0.291	
Widowed/Divorced/Separated	5,266 (28.12)	1.14 (1.08–1.19)	**<0.001**	
Never married	3,047 (16.27)	1.08 (0.95–1.23)	0.222	
Race				0.378
Mexican American	3,093 (16.52)	1.13 (1.01–1.26)	**0.032**	
Other Hispanic	1,568 (8.37)	1.05 (0.91–1.21)	0.512	
Non-Hispanic White	8,742 (46.68)	1.06 (1.01–1.1)	**0.012**	
Non-Hispanic Black	3,749 (20.02)	1.11 (1.03–1.19)	**0.007**	
Other race	1,574 (8.41)	1.17 (1.03–1.32)	**0.013**	
Hypertension				0.972
Yes	7,743 (41.35)	1.08 (1.04–1.13)	**<0.001**	
No	10,983 (58.65)	1.07 (1.01–1.13)	**0.016**	
DM				0.704
Yes	2,923 (15.61)	1.08 (1.02–1.15)	**0.014**	
No	15,803 (84.39)	1.08 (1.04–1.12)	**<0.001**	
Smoking status				0.858
At least 100 cigarettes in life	11,663 (62.28)	1.08 (1.03–1.13)	**<0.001**	
No/<100 cigarettes in life	7,063 (37.72)	1.08 (1.03–1.13)	**0.002**	
BMI				0.504
< 25 kg/m^2^	5,709 (30.49)	1.1 (1.04–1.17)	**<0.001**	
25–30 kg/m^2^	5,420 (28.94)	1.07 (1–1.14)	**0.036**	
≥30 kg/m^2^	7,597 (40.57)	1.06 (1–1.11)	**0.032**	
CVD				0.135
NO	17,444 (93.15)	1.09 (1.05–1.13)	**<0.001**	
YES	1,282 (6.85)	1.01 (0.93–1.1)	0.782	
Alcohol drinking				0.762
No/<12 cups per year	3,890 (20.77)	1.06 (0.99–1.13)	0.106	
At least 12 cups per year	14,836 (79.23)	1.08 (1.04–1.12)	**<0.001**	
Age				0.183
<48	9,212 (49.19)	0.99 (0.89–1.11)	0.9	
≥48	9,514 (50.81)	1.09 (1.05–1.13)	**<0.001**	

OR (95% CI): Odds Ratio with 95% Confidence Interval. P for interaction: Evaluates heterogeneity across subgroups using interaction terms in the regression model. Edu, Education; DM, Diabetes Mellitus; PIR, Poverty Income Ratio, CVD, Cardiovascular Disease, BMI, Body Mass Index. Bold values indicate statistically significant *p*-values (*p* < 0.05).

### Potential mechanistic associations

Using a fully adjusted model, the association between the NPAR and sex hormone biomarkers was investigated. A positive correlation was observed between the NPAR and T2 level [*β* = 48.40 (41.11–55.70), *p* < 0.001]. Similarly, a significant positive correlation [*β* = 3.03 (2.37–3.69), *p* < 0.001] was observed between the NPAR and SHBG levels. Furthermore, we evaluated the association between the NPAR and sex hormone levels in the control group to further eliminate the influence of BC patients in the overall population on the findings, and the findings were in line with those of the entire population (Table 4).

**Table 4:** Associations between NPAR and sex hormones.

Mediating factors	Overall		Controls	
	*β* (95% CI)	*p*-value	*β* (95% CI)	*p*-value
E2	0.12 (−0.15–0.38)	0.389	0.19 (−0.08–0.47)	0.169
T2	48.40 (41.11–55.70)	**<0.001**	50.07 (42.55–57.60)	**<0.001**
SHBG	3.03 (2.37–3.69)	**<0.001**	3.14 (2.47–3.82)	**<0.001**

The model was adjusted for age, education, PIR, marital status, race, hypertension, DM, smoking status, BMI, CVD and alcohol drinking. E2, estradiol; TT, total testosterone; SHBG, sex hormone-binding globulin. Bold values indicate statistically significant *p*-values (*p* < 0.05).

## Discussion

This is the first study that investigates, in a nationally representative population, whether the NPAR affects BC prevalence in women. We found that the prevalence of breast cancer increased with each unit increase in the NPAR, showing a linear correlation between the NPAR and BC prevalence. The reliability of our findings was validated using several stratified and sensitivity analyses; however, the findings showed that marital status may have an impact on the outcomes. Furthermore, another study has revealed that the NPAR may be influencing the occurrence of breast cancer by altering sex hormone levels. These findings highlight the significance of the NPAR as a putative biomarker for BC diagnosis in women.

Many inflammatory and nutritional markers have been used to evaluate BC prevalence. A retrospective analysis of hemoglobin–albumin–lymphocyte–platelet (HALP) scores and BC showed that a high HALP score is positively correlated with high overall survival and progression-free survival (14). Through sex hormone interference, the Advanced Lung Cancer Inflammatory Index (ALI) could decrease the incidence of BC, based on a retrospective study (15). The majority of measures that provide a thorough evaluation of inflammation and nutritional status are complex and challenging. However, the NPAR, a composite metric that assesses inflammation and nutrition, is easy to compute and performs well in the prediction of multiple types of diseases. For example, Deng’s retrospective analysis of 438 Kawasaki disease patients revealed that the NPAR and hemoglobin (HB) were independent risk factors for Intravenous immunoglobulin (IVIG) resistance (16). A prospective study showed that the NPAR may be a valuable biomarker for predicting spontaneous bacterial peritonitis (17). This study suggested that the clinical value of the NPAR needs to be further explored.

Chronic inflammation plays a crucial role in the development and progression of tumors. While the inflammatory response reflects the body’s defense mechanisms, prolonged exposure to inflammation may increase the risk of cancer (18). Studies have shown that patients with chronic pancreatitis have a significantly higher risk of developing pancreatic cancer compared to the general population (19). An inflammatory tumor microenvironment (TME) is created when tumor cells interact with the surrounding stromal cells and inflammatory immune cells, which accelerates the growth of the tumor (20). The reactive oxygen species (ROS) pathway is a key mechanism in the complicated processes through which chronic inflammation stimulates the development of tumors. ROS can either directly damage cellular DNA or indirectly activate cell-signaling pathways to promote metastasis and tumor progression (21). Furthermore, preclinical models (*in vitro* and *in vivo*) showed that pharmacological inhibition of COX-2 significantly suppresses the progression of breast tumor, which highlights its potential as a therapeutic strategy (22). This observation confirms that inflammation plays a key role in tumor development. Albumin is an important indicator of nutritional status, and in general, people with good nutritional status have higher albumin levels than those with poor nutritional status. In addition to indicating nutritional status, albumin levels also show correlations with immunological and inflammatory status (23). Utariani et al. reported that the high-protein, high-nutrition group had lower serum levels of inflammatory components (TNF-*α*, IL-1, and C-reactive protein (CRP)) than the low-protein group (24). This finding has been supported by several studies (25, 26). Furthermore, it has been demonstrated that albumin can reduce inflammation by blocking inflammatory mediators, including TNF-α and C5a (27, 28). Moreover, albumin has antioxidant properties (29), which can inhibit cancer progression (30, 31). These findings support our results that low nutritional status and increased inflammatory levels accelerate the onset and progression of BC.

Our findings showed that the levels of sex hormones are regulated by nutritional status and inflammatory levels, which in turn affects the development of BC. Sex hormones play a significant role in the multifactorial combination that leads to the development and progression of BC (32–34). Sex hormone levels are affected by chronic inflammation because it generates pro-inflammatory cells, such as TNF-*α* and IL-6, which activate the hypothalamic–pituitary–adrenal axis and promote the release of cortisol (35). The occurrence and progression of PCOS can be attributed to the imbalance in sex hormone levels caused by chronic inflammation (36, 37). Dysregulation of sex hormones promotes the onset and progress of cancer.

In this study, an association was observed between the NPAR and marital status in relation to BC risk. The underlying mechanisms may involve two pathways: (1) socio-contextual confounding pathways, where marital status indirectly modulates the NPAR and BC risk through lifestyle factors (e.g., dietary habits, medical adherence) and psychosocial stress, and (2) potential biological pathways, such as mediation via sex hormone fluctuations and chronic inflammation (although direct evidence remains lacking). However, due to the limitations of the cross-sectional design (inability to exclude reverse causation) and unmeasured key variables (e.g., marital quality, cortisol levels), current evidence predominantly supports marital status as a *socio-contextual confounder* that influences risk associations through non-biological mechanisms. Future studies should integrate multidimensional indicators (social–psychological–biological) and use a longitudinal design to clarify the specific mechanisms underlying this interaction.

This study showed a significant association between the NPAR and BC risk (OR = 1.05, 95% CI = 1.02–1.09, *p* = 0.003), with a higher risk observed in populations with an increased NPAR (e.g., the Q4 group, OR = 1.38). Although the effect size of the NPAR is lower than that of traditional genetic biomarkers such as BRCA mutations, its ease of use—based on routine blood tests and serum albumin assays—makes it a potential auxiliary tool for identifying high-risk individuals associated with inflammatory or malnutritional states, thereby optimizing screening strategies (e.g., reducing imaging follow-up intervals for high-risk groups). Future research should focus on validating NPAR’s predictive efficacy for BC incidence (rather than prevalence) by conducting prospective cohort studies, exploring models that take into account classical inflammatory markers (e.g., NLR, CRP), and designing intervention trials (e.g., anti-inflammatory therapies or nutritional support) to evaluate its preventive potential.

The NPAR, as a combined measure of inflammation and nutritional levels in the body, predicts the prognosis and progression of a wide range of diseases (12, 38). This study is the first study to investigate its relationship with BC prevalence. Our findings show that the NPAR may be a novel biomarker for BC. Monitoring the NPAR aids in the diagnosis of BC and can direct the development of nutritional plans and therapeutic medicines.

However, our study has some limitations. First, this study is inherently limited to revealing statistical associations between the NPAR and BC rather than establishing causality. Key limitations include residual confounding, reverse causation, and temporal ambiguity. Second, although we attempted to adjust for numerous covariates to mitigate confounding bias, we could not eliminate potential residual confounders such as socioeconomic status and cortisol levels, which were neither systematically measured nor excluded from the analysis. In addition, some variables that may influence inflammation and albumin levels, such as liver diseases, dietary intake, and medication use, were not controlled. Third, the majority of variables used in this study were self-reported through questionnaires, which might have introduced a recall bias. Furthermore, due to data constraints, we were unable to further explore the difference in statistical significance across NPAR quartiles and the nuanced relationship between the NPAR and marital status. Therefore, prospective cohort studies with rigorous biomarker measurements and a longitudinal design is warranted to validate these findings.

## Conclusion

Our findings demonstrated a positive association between breast cancer (BC) prevalence and the NPAR, potentially mediated by NPAR’s influence on sex hormone levels. The NPAR may serve as a biomarker for BC in adult US women, and reducing inflammation and maintaining proper nutrition may help lower the risk of BC.

## Data Availability

The datasets presented in this study can be found in online repositories. The names of the repository/repositories and accession number(s) can be found at: https://www.cdc.gov/nchs/nhanes/index.htm.
