# Supplementary material for: Association between neutrophil percentage-to-albumin ratio and breast cancer in adult women in the US: findings from the NHANES
Source: Front Nutr. 2025 Apr 28;12:1533636. doi: 10.3389/fnut.2025.1533636 (PMC12066505; doi:10.3389/fnut.2025.1533636)
Supplement: Supplementary file 1 [file Table_1.docx]

Table S1: Baseline characteristics of participants before and after PSM

| Variable | Before PSM | | | | | After PSM | | | | | |
| --- | --- | --- | --- | --- | --- | --- | --- | --- | --- | --- | --- |
|  | Total (n = 18726) | **Control** (n = 18215) | **Breast cancer** (n = 511) | *P* | SMD |  | Total (n = 1020) | **Control** (n = 510) | **Breast cancer** (n = 510) | *P* | SMD |
| **Age**, Mean ± SD | 49.04 ± 18.04 | 48.53 ± 17.92 | 67.21 ± 11.83 | **<.001** | 1.579 |  | 67.11 ± 12.13 | 67.05 ± 12.44 | 67.17 ± 11.81 | 0.873 | 0.010 |
| **Edu,** n (%) |  |  |  | 0.480 |  |  |  |  |  | 0.817 |  |
| Less than high school | 4353 (23.25) | 4245 (23.30) | 108 (21.14) |  | -0.053 |  | 220 (21.57) | 112 (21.96) | 108 (21.18) |  | -0.019 |
| High school or equivalent | 4191 (22.38) | 4077 (22.38) | 114 (22.31) |  | -0.002 |  | 234 (22.94) | 120 (23.53) | 114 (22.35) |  | -0.028 |
| College or above | 10182 (54.37) | 9893 (54.31) | 289 (56.56) |  | 0.045 |  | 566 (55.49) | 278 (54.51) | 288 (56.47) |  | 0.040 |
| **PIR**, n (%) |  |  |  | **<.001** |  |  |  |  |  | 0.794 |  |
| ≤1.30 | 5955 (31.8) | 5837 (32.05) | 118 (23.09) |  | -0.212 |  | 230 (22.55) | 112 (21.96) | 118 (23.14) |  | 0.028 |
| 1.30–3.50 | 7124 (38.04) | 6914 (37.96) | 210 (41.10) |  | 0.064 |  | 416 (40.78) | 206 (40.39) | 210 (41.18) |  | 0.016 |
| > 3.50 | 5647 (30.16) | 5464 (30.00) | 183 (35.81) |  | 0.121 |  | 374 (36.67) | 192 (37.65) | 182 (35.69) |  | -0.041 |
| **Marital status**, n (%) |  |  |  | **<.001** |  |  |  |  |  | 0.321 |  |
| Married/Living with partner | 10413 (55.61) | 10156 (55.76) | 257 (50.29) |  | -0.109 |  | 494 (48.43) | 238 (46.67) | 256 (50.20) |  | 0.071 |
| Widowed/Divorced/Separated | 5266 (28.12) | 5039 (27.66) | 227 (44.42) |  | 0.337 |  | 477 (46.76) | 250 (49.02) | 227 (44.51) |  | -0.091 |
| Never married | 3047 (16.27) | 3020 (16.58) | 27 (5.28) |  | -0.505 |  | 49 (4.8) | 22 (4.31) | 27 (5.29) |  | 0.044 |
| **Race,** n (%) |  |  |  | **<.001** |  |  |  |  |  | 0.636 |  |
| Mexican American | 3093 (16.52) | 3052 (16.76) | 41 (8.02) |  | -0.321 |  | 73 (7.16) | 32 (6.27) | 41 (8.04) |  | 0.065 |
| Other Hispanic | 1568 (8.37) | 1539 (8.45) | 29 (5.68) |  | -0.120 |  | 55 (5.39) | 26 (5.10) | 29 (5.69) |  | 0.025 |
| Non-Hispanic White | 8742 (46.68) | 8406 (46.15) | 336 (65.75) |  | 0.413 |  | 690 (67.65) | 355 (69.61) | 335 (65.69) |  | -0.083 |
| Non-Hispanic Black | 3749 (20.02) | 3674 (20.17) | 75 (14.68) |  | -0.155 |  | 148 (14.51) | 73 (14.31) | 75 (14.71) |  | 0.011 |
| Other Race | 1574 (8.41) | 1544 (8.48) | 30 (5.87) |  | -0.111 |  | 54 (5.29) | 24 (4.71) | 30 (5.88) |  | 0.050 |
| **Hypertension**, n (%) |  |  |  | **<.001** |  |  |  |  |  | 0.514 |  |
| YES | 7743 (41.35) | 7411 (40.69) | 332 (64.97) |  | 0.509 |  | 654 (64.12) | 322 (63.14) | 332 (65.10) |  | 0.041 |
| NO | 10983 (58.65) | 10804 (59.31) | 179 (35.03) |  | -0.509 |  | 366 (35.88) | 188 (36.86) | 178 (34.90) |  | -0.041 |
| **DM**, n (%) |  |  |  | **<.001** |  |  |  |  |  | 0.476 |  |
| YES | 2923 (15.61) | 2785 (15.29) | 138 (27.01) |  | 0.264 |  | 266 (26.08) | 128 (25.10) | 138 (27.06) |  | 0.044 |
| NO | 15803 (84.39) | 15430 (84.71) | 373 (72.99) |  | -0.264 |  | 754 (73.92) | 382 (74.90) | 372 (72.94) |  | -0.044 |
| **Smoking status,**n (%) |  |  |  | **0.025** |  |  |  |  |  | 0.527 |  |
| At least 100 cigarettes in life | 11663 (62.28) | 11369 (62.42) | 294 (57.53) |  | -0.099 |  | 578 (56.67) | 284 (55.69) | 294 (57.65) |  | 0.040 |
| NO/ Less than 100 cigarettes in life | 7063 (37.72) | 6846 (37.58) | 217 (42.47) |  | 0.099 |  | 442 (43.33) | 226 (44.31) | 216 (42.35) |  | -0.040 |
| **BMI**, n (%) |  |  |  | 0.642 |  |  |  |  |  | 0.675 |  |
| < 25 kg/m^2^ | 5709 (30.49) | 5548 (30.46) | 161 (31.51) |  | 0.023 |  | 312 (30.59) | 151 (29.61) | 161 (31.57) |  | 0.042 |
| 25-30 kg/m^2^ | 5420 (28.94) | 5267 (28.92) | 153 (29.94) |  | 0.022 |  | 316 (30.98) | 164 (32.16) | 152 (29.80) |  | -0.051 |
| ≥ 30 kg/m^2^ | 7597 (40.57) | 7400 (40.63) | 197 (38.55) |  | -0.043 |  | 392 (38.43) | 195 (38.24) | 197 (38.63) |  | 0.008 |
| **CVD**, n (%) |  |  |  | **<.001** |  |  |  |  |  | 0.309 |  |
| NO | 17444 (93.15) | 17007 (93.37) | 437 (85.52) |  | -0.223 |  | 885 (86.76) | 448 (87.84) | 437 (85.69) |  | -0.062 |
| YES | 1282 (6.85) | 1208 (6.63) | 74 (14.48) |  | 0.223 |  | 135 (13.24) | 62 (12.16) | 73 (14.31) |  | 0.062 |
| **Alcohol drinking**, n (%) |  |  |  | 0.592 |  |  |  |  |  | 0.412 |  |
| NO/ Less than 12 cups per year | 3890 (20.77) | 3779 (20.75) | 111 (21.72) |  | 0.024 |  | 233 (22.84) | 122 (23.92) | 111 (21.76) |  | -0.052 |
| At least 12 cups per year | 14836 (79.23) | 14436 (79.25) | 400 (78.28) |  | -0.024 |  | 787 (77.16) | 388 (76.08) | 399 (78.24) |  | 0.052 |
| **NPAR, Mean ± SD** | 14.38 ± 3.03 | 14.37 ± 3.04 | 14.71 ± 2.81 | **0.013** | 0.120 |  | 14.37 ± 2.73 | 14.04 ± 2.61 | 14.71 ± 2.81 | **<.001** | 0.240 |

SMD: Standard Mean Diddirence.

Abbreviation: Edu:Education; DM:Diabetes Mellitus; PIR:Poverty Income Ratio; CVD:Cardiovascular Disease; BMI:Body Mass Index.
